# Application of White Mustard Bran and Flour on Bread as Natural Preservative Agents

**DOI:** 10.3390/foods10020431

**Published:** 2021-02-16

**Authors:** Raquel Torrijos, Tiago de Melo Nazareth, Juan Manuel Quiles, Jordi Mañes, Giuseppe Meca

**Affiliations:** Department of Food Chemistry and Toxicology, Faculty of Pharmacy, University of Valencia, Ave. Vicent Andrés Estellés s/n, 46100 Burjassot, Spain; raquel.torrijos@uv.es (R.T.); juan.quiles@uv.es (J.M.Q.); jorge.manes@uv.es (J.M.); giuseppe.meca@uv.es (G.M.)

**Keywords:** mustard bran, by-product, antifungal, bakery products, shelf-life, *Sinapis alba*, mustard flour, food safety, mycotoxigenic fungi, mycotoxins

## Abstract

In this study, the antifungal activity of white mustard bran (MB), a by-product of mustard (*Sinapis alba*) milling, and white mustard seed flour (MF) was tested against mycotoxigenic fungi in the agar diffusion method. The results obtained were posteriorly confirmed in a quantitative test, determining the minimum concentration of extract that inhibits the fungal growth (MIC) and the minimum concentration with fungicidal activity (MFC). Since MF demonstrated no antifungal activity, the MB was stored under different temperature conditions and storage time to determine its antifungal stability. Finally, an in situ assay was carried out, applying the MB as a natural ingredient into the dough to avoid *P. commune* CECT 20767 growth and increase the bread shelf life. The results demonstrated that the antifungal activity of MB was dose-dependent. The higher assayed dose of MB (10 g/kg) reduced the fungal population in 4.20 Log CFU/g regarding the control group. Moreover, the shelf life was extended four days compared to the control, equaling its effectiveness with the synthetic preservative sodium propionate (E-281). Therefore, MB could be an alternative to chemical additives in bread formulations since it satisfies consumer requirements. Also, the formulation of bread with MB valorizes this by-product generated during mustard seed milling, thereby helping the industry move forward sustainably by reducing environmental impact.

## 1. Introduction

Bakery products are subject to various spoilage problems, such as chemical, physical, and microbial [1]. Bacteria, fungi, and yeast can cause spoilage of bread due to its relatively high-water activity and alkaline pH. These properties are suitable for the growth of a wide range of fungal species. Indeed, fungal spoilage is the primary concern in the bakery industry. Contamination occurs predominantly after baking, by fungal spores from the environment that settle on food [2]. The genera *Penicillium* and *Aspergillus* are the most common fungi responsible for the deterioration of bread [3].

In addition to the fungal spoilage, these microorganisms can also produce mycotoxins [4,5,6]. Mycotoxins are highly toxic metabolites produced by various fungal species in certain foods, including bread. Among them, Zearalenone, Ochratoxin A, and Aflatoxin B1 are mycotoxin with significant occurrence in wheat and wheat products worldwide [7,8,9,10,11]. These molecules are of concern to human health because of their toxicological properties and the capacity to maintain food even after thermal processing. For instance, Aflatoxin B1, Ochratoxin A, and Zearalenone have been associated with hepatotoxicity, nephrotoxicity, and hyperestrogenism [12,13]. Besides, based on recent reports, mycotoxin prevalence could be as high as 60–80% in food [4,14].

The consumption of bakery products has changed in recent decades due to the growing alert of consumers about health and environmental issues that have led to an increase in demand for whole food and food products without chemical additives or preservatives [15]. Accordingly, new products have been introduced to the market, and food preservation has become increasingly complex. These products required more extended shelf life and a more generous guarantee of the absence of foodborne pathogenic organisms, as well as a reduction in the application of chemical preservatives [16]. Such requirements offered new challenges and opportunities for those who have sought new food preservatives.

Nowadays, the overuse of chemical additives has increased the pressure on food manufacturers to remove chemical preservatives from their food products, some of which are suspected of their alleged or toxicity potential, and adopt natural alternatives to maintaining or extending the shelf life of a product [17]. Against this background, natural origin substances may be an excellent alternative for preserving these products since the search for new chemical substances is hampered by regulatory restrictions; hence, much time and money may be needed to develop a newly approved preservative that satisfies the public pressure against chemical additives.

White mustard (Sinapis alba) belongs to the Brassicaceae family. These plants are known for the high concentration of glucosinolates, which play an important role in plant defense. A plant injury activates the glucosinolate–myrosinase system, leading to enzymatic hydrolysis of glucosinolates by myrosinase. This reaction culminates in isothiocyanates (ITC) that have been recognized for their human nutrition benefits [18]. The ITC are also molecules with a known fungicidal, bactericidal, and insecticidal activity that stand out for their antifungal effects [19,20,21,22]. In white mustard, the hydrolysis of the glucosinolate sinalbin generates the ITC p-hydroxybenzyl isothiocyanate (p-HBIT), which has significant antimicrobial activity against bacteria and fungi [23].

The commercial preparation of mustard seed flour (MF) produces many by-products, called mustard bran (MB). The MB is the outer husk removed from mustard seed during milling. On the one hand, MB represents a valuable by-product because it is rich in proteins (16 wt%), hydrophilic polysaccharides, high-molecular-weight polysaccharides, and a significant antioxidant activity provided by uronic acid [24]. On the other hand, the MB is costly to eliminate and could be deleterious to the environment if discharged in wastewater; therefore, using MB as a natural antifungal ingredient can be an alternative to alleviate this problem.

According to current knowledge, the application of mustard by-products as an antifungal compound was not studied. Therefore, the objective of this study was to evaluate the antifungal potential of MF and MB against toxigenic strains of the genera *Aspergillus*, *Fusarium*, and *Penicillium*. Accordingly, we proposed a method of extraction of water-soluble components to assess their antifungal activities in vitro. Furthermore, we determined the antifungal compound stability by incubating the extracts at different temperatures and storage times. Finally, MB was employed in the dough formulation to increase the shelf life and to avoid the growth of *P. commune* CECT 20767 on the bread.

## 2. Materials and Methods

### 2.1. Chemicals

The microbiological products such as Potato Dextrose Broth (PDB), Potato Dextrose Agar (PDA), and Buffered Peptone water were obtained from Liofilchem Bacteriology Products (Roseto, Italy). The deionized water was obtained from a Milli-Q water purification system. The TWEEN^®^ 80 and the sodium propionate (E-281) were obtained from Sigma-Aldrich (St. Louis, MO, USA). The white mustard seed (product code 501) and the white MB (product code 412) were purchased from G. S. Dunn (Hamilton, ON, Canada).

### 2.2. Fungal Strains and Culture Conditions

The toxigenic fungal strains *P. camemberti* CECT 2267, *P. expansum* CECT 2278, *P. roqueforti* CECT 2905, *P. digitatum* CECT 2954, *P. commune* CECT 20767, *P. solitum* CECT 20818, *A. parasiticus* CECT 2681, *A. ochraceus* CECT 2093, *A. lacticoffeatus* CECT 20581, *A. steynii* CECT 20510, *A. tubingensis* CECT 20543 and *A. tubingensis* CECT 20544 were purchased from the Spanish Type Culture Collection CECT (Valencia, Spain). *A. flavus* ITEM 8111, *F. verticillioides* ITEM 12052, *F. proliferatum* ITEM 12072, *F. verticillioides* ITEM 12044, *F. graminearum* ITEM 126, *F. sporotrichioides* ITEM 12168, and *F. poae* ITEM 9151 were obtained from the Institute of Sciences of Food Production ISPA (Bari, Italy). The strain *P. verrucosum* VTT D-01847 was purchased from the VTT Technical Research Centre of Finland LTD (Otaniemi, Finland). The fungal strains were preserved in liquid PDB with 30% glycerol. Then, the strains were thawed and inoculated in PDB medium for one week at 25 °C. Afterward, the mycelium was transferred to a PDA plate and incubated again for five days at 25 °C. These PDA plates were used to harvest the fungal spores employed in this study.

### 2.3. Extraction of Water-Soluble Mustard Components

The mustard seed was ground using an Oster classic grinder (Valencia, Spain) before the extraction step. The extraction of water-soluble components was realized as follows. MF or MB (4 g) were homogenized with 50 mL of distilled water using an Ultra Ika T18 basic UltraTurrax (Staufen, Germany) for 5 min at 12,000× *g*. Next, the extracts were centrifuged for 15 min at 5000× *g*, and the supernatant was recovered and placed in polypropylene trays. Posteriorly, the supernatant was freeze-dried in a FreeZone 2.5 L Labconco (Kansas, MO, USA) for 72 h. The powder obtained was stored at −20 °C before use in the antifungal activity tests.

### 2.4. Antifungal Activity of Freeze-Dried Mustard Extracts in Agar Diffusion Method

The freeze-dried powder of MF and MB was resuspended in sterile water at 100 g/L and tested against the toxigenic *Penicillium*, *Aspergillus*, and *Fusarium* strains described in item 2.2. For this, the fungal strains were sowed on PDA plates with a cotton swab soaked with sterile water TWEEN 0.2%. Next, a 10 mm well was realized on the PDA, and 100 μL of either MF or MB suspension was placed. The plates were incubated for 48 h at 25 °C, and then, the inhibition halos were measured on a scale of mm. The inhibition halos were classified as follows: (−) means that no inhibition halo was detected; (+) means that inhibition halos of 5 mm diameter were detected on PDA plates; (++) means that inhibition halos between 5–10 mm diameter were detected on PDA plates; and (+++) means that inhibition halos were more extensive than 10 mm diameter.

### 2.5. Antifungal Properties of MB over Time in Agar Diffusion Method

The antifungal stability of MB was studied, suspending the freeze-dried powder in sterile water at 100 g/L and then stored at three temperatures (4, 25, and 50 °C). The assay was performed during different storage times (24 h, 48 h, 72 h, and 168 h) using the same methodology described in Section 2.4. After 48 h of incubation at 25 °C, the inhibition halos were measured.

### 2.6. Determination of the Minimum Inhibitory Concentration (MIC) and the Minimum Fungicidal Concentration (MFC) of MB

The in vitro antifungal activity of MB was determined according to the CLSI document M38-A2 in 96-well microplates with minor modifications [25]. First, conidia concentration was measured and adjusted in PDB media to 5 × 10^4^ conidia/mL with a Neubauer chamber (Marienfeld, Lauda-Königshofen, DE). Then, the 96-well microplate was filled as follows. A negative control containing only PDB medium and a positive control containing PDB with the fungal strains were realized. The following columns were filled with 100 μL of freeze-dried MB resuspended in PDB at concentration decreasing from 100 to 0.4 g/L and 100 μL of the fungal strains. The 96-well microplates were incubated at 25 °C for 72 h. After the incubation time, the MIC value was established as the lowest MB concentration that visually inhibited the fungal growth. To determine the MFC value, 10 μL of the MICs higher doses were subcultured on PDA plates and incubated again at 25 °C for 72 h. The MFC was defined as the lowest extract concentration in which the fungal growth was prevented.

### 2.7. Application of MB in Bread Formulation

The bread dough (1 kg of weight) was prepared by mixing the following ingredients: 600 g of wheat flour, 20 g of sugar, 10 g of NaCl, 40 g of yeast (purchased from a local supermarket), and 250 mL of water. Four different MB doses were tested: 2.5 g/kg; 5 g/kg; 7.5 g/kg; and 10 g/kg. Commercial treatment was prepared with the additive sodium propionate (E-281) at a 2 g/kg concentration. The control treatment was realized following the recipe without including MB or chemical additives in its composition. The kneading was performed using a SilverCrest Bread Maker SBB 850 A1 (Kompernass GMBH, Bochum, Germany) for 10 min. The dough obtained was fermented for 1 h at room temperature (25 °C). The dough was then transferred to a cast and baked in a MIWE deck oven (Arnstein, Germany) at 200 °C for 40 min. The bread was cooled at room temperature in a sterile cabinet before sliced (30 g for each portion). After that, each bread-loaf sample was individually inoculated in nine equidistant points with 10 µL of a *P. commune* CECT 20767 solution adjusted to 3 × 10^5^ conidia/mL using a pipette. The loaves were placed in polyethylene bags and stored for seven days at room temperature.

### 2.8. Determination of the Fungal Population and Shelf Life

After seven days of incubation, the FP in bread loaves was evaluated according to Pitt & Hocking (1997) [26]. Each loaf (30 g) was placed in a sterile plastic bag containing 270 mL of sterile peptone water 0.1% and was homogenized in a Stomacher IUL (Barcelona, Spain) for 30 s. Then, an aliquot of 1 mL was tenfold diluted in Falcon tubes with sterile peptone water 0.1%, and 100 μL of each dilution was placed on PDA plates and spread with a Drigalski-hook. The plates were incubated for 48 h at 25 °C, and the fungal colonies were counted. The sampling was realized in duplicate.

The microbial shelf life was determined, according to Doulia et al. (2009) [27]. The contaminated loaves were visually analyzed daily. Shelf life ended when the growth of *P. commune* was detected on the surface because consumers would reject the product at the first sign of spoilage.

### 2.9. Statistical Analysis

GraphPad Prism version 3.0 software was employed for statistical analysis. The differences between treatments considering *p* < 0.05 were analyzed by a one-way-ANOVA statistical test followed by the Tukey post hoc test for multiple comparisons.

## 3. Results and Discussion

### 3.1. In Vitro Antifungal Activity of MB and Antifungal Stability over Time

The antifungal properties of the aqueous MF and MB extracts at 100 g/L were evaluated on PDA plates against toxigenic fungi of the *Aspergillus*, *Penicillium*, and *Fusarium* genera (Table 1). The MF extract only showed antifungal activity against *F. verticillioides* ITEM 12044 and *F. poae* ITEM 9151, with inhibition halos of 5 mm diameter. In contrast, the MB extract showed antifungal activity against all the strains tested, obtaining inhibition halos larger than 10 mm diameter for the *P. camemberti* CECT 2267, *P. expansum* CECT 2278, *P. roqueforti* CECT 2905, *P. commune* CECT 20767, *P. verrucosum* VTT D-01847, *F. verticillioides* ITEM 12052, and *F. verticillioides* ITEM 12044 strains. Despite MB has shown a significant antifungal potential towards *Aspergillus* spp., the *Aspergillus* strains demonstrated more resistance to the MB extract than the other genera, with inhibition halos lower than 5 mm in diameter.

The MB showed greater effectiveness in avoiding the toxigenic fungi growth in agar diffusion assay; thus, the following test was carried out to determine the stability of the antifungal compounds present in these extracts. In other words, the extracts were reevaluated through the agar diffusion method after incubation at different temperatures and storage times. Specifically, between 4 and 50 °C for 24–168 h. The data obtained are plotted in Table 2. The results suggested that the storage temperature directly affects the MB extract antifungal properties since the extract stored at 50 °C lost the antifungal activity after 24 h of storage. The extract stored at 25 °C also reduced their antifungal properties; however, even after 168 h of storage showed inhibition halos of 5 mm diameter against *Penicillium* and *Fusarium* genera.

The MB extract stored at 4 °C showed the highest antifungal activity during storage for all the fungi tested. In particular, the MB extract antifungal properties were preserved until 168 h of storage against *Penicillium* and *Fusarium* strains tested, showing inhibition halos between 5–10 mm in diameter. Thus, the temperature was a crucial factor in preserving the antifungal properties of MB extracts during storage. These findings may be explained by the myrosinase enzyme activity responsible for converting the sinalbin into P-HBIT. It has been reported that myrosinase activity in white mustard decreases significantly with temperatures above 60 °C for 10 min of exposure [28,29]. In our study, the higher time of exposure (24 h) for the MB extract to the heat treatment (50 °C) may explain the loss of the myrosinase activity and the loss of antifungal properties. Tsao et al. (2000) [30] also described that the stability of glucosinolates and the degradation rate of isothiocyanates are closely related to the pH of the aqueous solution, and they evidenced less stability when the medium was alkalized (pH 9.00). Therefore, it is worthy to note that the P-HBIT stability may differ depending on the extraction matrix because it interacts with other components that increase or decrease its stability over time.

In addition to the agar diffusion assay, we performed a quantitative test on the antifungal effectiveness of MB extracts, and the results are shown in Table 3. Two parameters were established: the Minimum Inhibitory Concentration (MIC) and the Minimum Fungicidal Concentration (MFC). The effectiveness of MB depended on the fungal species tested. *Penicillium* strains obtained the lower MIC values, with values ranging from 0.3 to 1.2 g/L; i.e., *Penicillium* spp. were more susceptible to MB exposure. The other fungal genera presented MIC values ranging from 0.6 to 2.3 g/L and 0.6 to 4.7 g/L for the *Aspergillus* and *Fusarium* genera, respectively. Regarding the MFC, the *Penicillium* strains also presented lower values, ranging from 0.6 to 4.7 g/L. It seems that MB water extract could be an alternative to control *Penicillium* spoilages in long term storage foods.

The most sensitive strains exposed to MB extract were *P. camemberti* CECT 2267 and *P. roqueforti* CECT 2905, with MFC values of 0.6 g/L. The *Fusarium* strains presented MFC values ranging from 2.3 to 9.4 g/L, and the *Aspergillus* strains MFC values ranging from 1.2 to 18.8 g/L. The strain with the most resistance properties to the MB extract was *A. tubingensis* CECT 20544, and a concentration of 18.8 g/L was needed to obtain the fungicidal effect. These results were consistent with those obtained on the agar diffusion method, evidencing, for the first time, a significant fungicidal effect of MB.

Previous studies have shown the efficacy of yellow mustard (*Sinapis alba*) and oriental mustard (*Brassica juncea*) to control toxigenic bacteria and fungi in vitro. These botanical antimicrobial activities were closely related to the production of ITC, which have been described in the literature as antimicrobial and antifungal agents [31,32]. Wang et al. (2020) [33] studied the antifungal properties of benzyl isothiocyanate (0.312, 0.625, and 1.25 mM) against the fungal pathogen *Alternaria alternata*. The authors noticed that that compound reduced the spore germination, the rate of mycelial growth, and the synthesis of *Alternaria* mycotoxins in a dose-dependent manner. Moreover, they proposed that the antifungal toxicity of BITC occurred through a cell growth hindrance and a membrane disruption. Azaiez et al. (2013) [34] studied the antifungal activity of allyl, benzyl, and phenyl isothiocyanate standard solutions prepared at 10, 25, and 50 μg/per plate against *Gibberella moniliformis*, a Fumonisin B1 producer, and monitored the mycelial growth. The employed ITC reduced the mycelial growth between 2.1–89.7% according to the dose and exposure time.

Even though the effectiveness of essential oils (EOs) in these previous studies was high, it is essential to emphasize that the application of EOs in foods is limited due to their intense residual flavors and odors [35]. The main advantage of MB in comparison with EOs is -in addition to using a by-product that would contaminate the environment- the high antifungal activity in aqueous solution, which facilitates its application as an antifungal agent in bakery product formulations.

### 3.2. Antifungal Properties of White Mustard Bran on Bread Formulations

**Figure 1:**
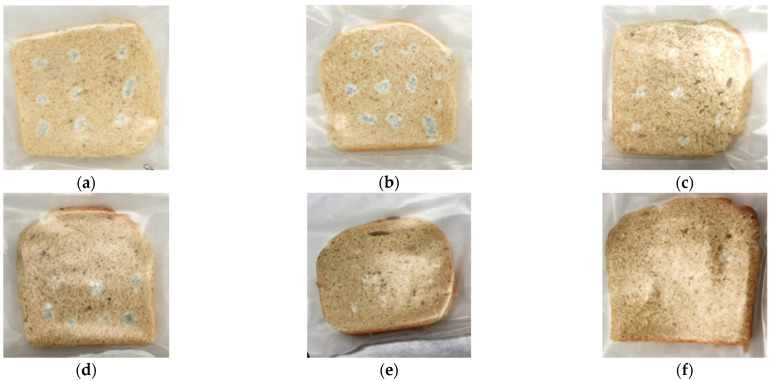
Bread slices contaminated with *P. commune* CECT 20767 after seven days of incubation at room temperature. Different treatments were carried out: (**a**) Control treatment; (**b**) 2.5 g/kg of white mustard bran (MB); (**c**) 5 g/kg of MB; (**d**) 7.5 g/kg of MB; (**e**) 10 g/kg of MB, and (**f**) Commercial treatment with sodium propionate (E-281).

The MB aqueous extract demonstrated a high antifungal effect in vitro, inhibiting the growth of toxigenic fungi that commonly affect food. Thus, we proposed the direct application of MB in the formulation of bread to enhance the shelf life. The fungal strain selected for this test was *P. commune* CECT 20767 because it is one of the primary contaminants isolated in bakery products [36]. Also, the MB extract showed a higher fungicidal effect against *Penicillium* strains. As a result, incorporating MB in the baking process increased the bread-slices shelf life in a dose-dependent manner (Table 4). In other words, MB reduced the *P. commune* CECT 20767 growth in a dose-dependent manner. For example, the MB dose at 2.5 g/kg did not increase the bread shelf life because the fungal growth was observed on the slice surface after four inoculation days. This result was similar to the control treatment that did not receive any preservatives. In contrast, the application of 5 and 7.5 g/kg of MB into the bread formulation expanded the shelf life by two days compared to the control treatment. The highest dose tested (10 g/kg) increased the shelf life of the bread slices up to day 7 of incubation, equaling the antifungal effect with the application of the synthetic chemical preservative sodium propionate (E-281) (Figure 1).

**Figure 2:**
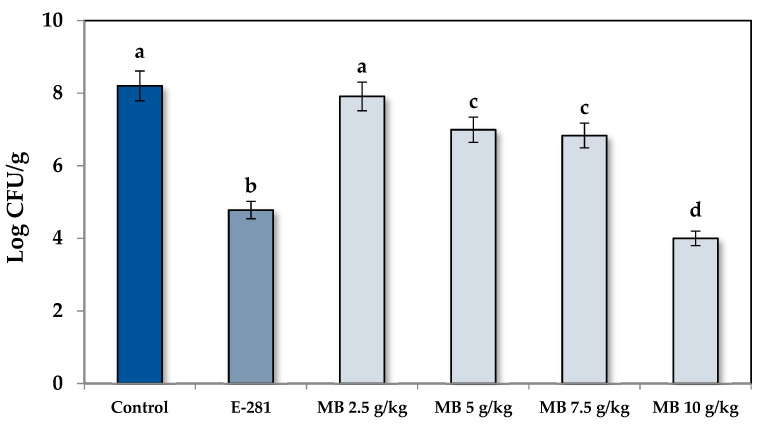
The fungal population of the loaf bread formulated with MB (2.5, 5, 7.5, and 10 g/kg) and contaminated with *P. commune* CECT 20767. The control group did not receive any treatment, and the commercial treatment was performed with sodium propionate (E-281) at 2 g/kg. Different letters mean significant differences between treatments (*p* < 0.05).

Regarding the fungal population (FP), the shelf life results were corroborated by counting the fungal colonies of the bread slices at day 7 of incubation. Likewise, as plotted in Figure 2, the bread elaborated with MB at 2.5 g/kg did not significantly reduce the FP regarding the control treatment (*p* < 0.05). However, statistically, significant differences were observed for MB at 5 g/kg and 7.5 g/kg. Specifically, the FP was reduced by 1.21 and 1.37 log CFU/g compared to the control treatment, which meant a reduction of 96.1 and 97.3%, respectively. The most effective dose was 10 g/kg of MB, which reduced the FP by 4.20 Log CFU/g (99.9% reduction), and it also equaled the effectiveness of the treatment with additive E-281. The data obtained showed that MB is a potential candidate for a preservative ingredient in bakery products affected by the genus *Penicillium*. Therefore, its incorporation into the bread dough could avoid fungal growth and, consequently, increase the shelf life and the safety of these foods.

The use of mustard-derived isothiocyanates was previously applied to reduce the growth of pathogenic microorganisms in different food systems. These studies focused on preventing fungal growth in bakery products, avoiding mycotoxin production, and improving bread shelf life. Saladino et al. (2016) [37] studied the mycotoxin reduction of patulin in wheat tortillas using volatile bioactive compounds released from oriental and white mustard flour. The results showed that 0.5, 1.0, and 2.0 g of both mustard flour released ITC in the samples, but no significant differences were detected in the fungal population. Nonetheless, the authors achieved a higher percentage of patulin reduction than 80%.

Quiles et al. (2015) [38] also studied the inhibition of *A. parasiticus* and aflatoxin reduction in pizza crust using allyl isothiocyanate by multiple strategies. The active packaging strategy consisted of applying allyl isothiocyanate (AITC) at final concentrations of 2, 5, and 10 μL/L through a sachet or a paper filter. The authors also evaluated the inclusion of autoclaved oriental mustard into the dough (0.7, 1.7, or 3.4% as dry ingredients). The authors showed that the autoclaved oriental mustard flour did not reduce the *A. parasiticus* growth and aflatoxin production. The difference between that study and our results may be explained because the authors utilized another variety of mustard (*Brassica juncea*), and we also applied MB into the dough without previous heat treatment. Hence, the activity of the myrosinase enzyme remained intact. Notwithstanding the preceding, the active packaging strategies proposed were effective in a dose-dependent manner.

Furthermore, Clemente et al. (2019) [39] evaluated the antifungal activity of mustard EO on commercial traditional Spanish bread contaminated with *Rhizopus stolonifer*. The bread was treated with 1 μL of EO (AITC purity > 95%) and incubated at two temperatures (25 and 4 °C). They evidenced that the antifungal activity of AITC reduced in the food matrix compared to in vitro studies; however, the fungicidal effect was significant to improve the shelf life in both storage temperatures.

Despite the application of essential mustard oils, allyl isothiocyanate and p-hydroxybenzyl isothiocyanate had previously been studied as effective strategies to reduce toxigenic microorganisms in food; nowadays, there are no reports on the MB application as an antifungal agent. This novel approach allowed one to employ a by-product that increases the shelf life of bread. Moreover, it is worth highlighting that MB satisfies the consumer demand for natural products, either avoiding or reducing the application of traditional synthetic chemical additives. Against this background and adding the results obtained in our study, it can be concluded that white-mustard bran could be a strategy of great interest because it delays the spoilage caused by mycotoxigenic and non-mycotoxigenic fungi and increases the shelf life of bakery products, reducing economic losses. Furthermore, the incorporation of MB into the dough could reduce environmental waste.

## 4. Conclusions

In the present study, the MB, a by-product of yellow mustard (*Sinapis alba*), was proposed as an antifungal agent. The evaluation of the aqueous extracts in vitro against the toxigenic fungi genera *Aspergillus*, *Fusarium*, and *Penicillium* confirmed the antifungal activity of the MB and its stability during storage at 4 and 25 °C. MB was also evaluated as an enhancer of the microbiological shelf life of bread contaminated with *P. commune* CECT 20767, a traditional spoilage agent of bakery products.

The application of 10 g/kg of MB resulted in an effective strategy to reduce the fungal contamination without compromising the organoleptic properties. Moreover, 10 g/kg doses also obtained similar results to the sodium propionate regarding the bread shelf-life. For this reason, MB could be a good candidate to replace traditional preservatives as sodium propionate once MB could satisfy consumer demand by reducing the content of synthetic chemical additives in food.

Furthermore, it is important to note that MB is a cheap ingredient priced at around $1.40/kg, which could lead to cheaper bakery products.

Further research will focus on applying MB in commercial bakery products associated with different storage conditions, such as vacuum and modified atmosphere packaging. Likewise, the identification of MB antifungal compounds and the direct application of MB extracts will be studied.

## Figures and Tables

**Table 1:** Antifungal activity of white mustard seed flour (MF) and white mustard bran (MB) extracts in agar diffusion method against *Penicillium*, *Aspergillus*, and *Fusarium* spp.

Fungi Strain	MF	MB
*P. camemberti* CECT 2267	−	+++
*P. expansum* CECT 2278	−	+++
*P. roqueforti* CECT 2905	−	+++
*P. digitatum* CECT 2954	−	++
*P. commune* CECT 20767	−	+++
*P. solitum* CECT 20818	−	++
*P. verrucosum* VTT D-01847	−	+++
*A. flavus* ITEM 8111	−	+
*A. parasiticus* CECT 2681	−	+
*A. ochraceus* CECT 2093	−	+
*A. lacticoffeatus* CECT 20581	−	+
*A. steynii* CECT 20510	−	+
*A. tubingensis* CECT 20543	−	+
*A. tubingensis* CECT 20544	−	+
*F. proliferatum* ITEM 12072	−	++
*F. verticillioides* ITEM 12052	−	+++
*F. verticillioides* ITEM 12044	+	+++
*F. graminearum* ITEM 126	−	++
*F. sporotrichioides* ITEM 12168	−	+
*F. poae* ITEM 9151	+	++

The inhibition halos were measured on an mm scale: (−) means no inhibition halo detected; (+) means inhibition halo of 5 mm diameter; (++) means inhibition halo ≥5 mm and <10 mm diameter; and (+++) means inhibition halo ≥10 mm diameter.

**Table 2:** White mustard bran (MB) antifungal stability test during storage at different temperatures (4, 25, and 50 °C). The extract was prepared at 100 g/L and tested against toxigenic *Aspergillus*, *Fusarium*, and *Penicillium* strains.

Fungi Strain	4 °C				25 °C				50 °C
	24 h	48 h	72 h	168 h	24 h	48 h	72 h	168 h	24–168 h
*P. camemberti* CECT 2267	+++	+++	+++	++	++	++	++	+	−
*P. expansum* CECT 2278	+++	+++	+++	++	++	++	++	+	−
*P. roqueforti* CECT 2905	+++	+++	+++	++	++	++	++	+	−
*P. digitatum* CECT 2954	+++	+++	+++	++	++	++	+	+	−
*P. commune* CECT 20767	+++	+++	+++	++	++	++	++	+	−
*P. solitum* CECT 20818	+++	+++	+++	++	++	++	++	+	−
*P. verrucosum* VTT D-01847	+++	+++	+++	++	++	++	++	++	−
*A. flavus* ITEM 8111	+	+	+	−	+	+	−	−	−
*A. parasiticus* CECT 2681	+	+	+	−	+	+	−	−	−
*A. ochraceus* CECT 2093	+	+	+	−	+	+	−	−	−
*A. lacticoffeatus* CECT 20581	+	+	+	−	+	+	−	−	−
*A. steynii* CECT 20510	+	+	+	−	+	+	−	−	−
*A. tubingensis* CECT 20543	+	+	+	−	+	+	−	−	−
*A. tubingensis* CECT 20544	+	+	+	−	+	+	−	−	−
*F. proliferatum* ITEM 12072	++	++	++	+	++	++	++	+	−
*F. verticillioides* ITEM 12052	+++	+++	+++	++	++	++	++	+	−
*F. verticillioides* ITEM 12044	+++	+++	++	+	++	++	+	+	−
*F. graminearum* ITEM 126	++	++	++	+	+	+	+	+	−
*F. sporotrichioides* ITEM 12168	+	+	+	+	+	+	+	+	−
*F. poae* ITEM 9151	++	++	++	+	++	++	++	+	−

The inhibition halos were measured on an mm scale as follows: (−) means no inhibition halo detected; (+) means inhibition halo of <5 mm diameter; (++) means inhibition halo ≥5 mm and <10 mm diameter; and (+++) means inhibition halo ≥10 mm diameter.

**Table 3:** Minimum Inhibitory Concentration (MIC) and Minimum Fungicidal Concentration (MFC) of white mustard bran extract (MB) evaluated against *Penicillium*, *Aspergillus*, and *Fusarium* strains. Results are expressed in g/L.

Fungi Strain	MIC	MFC
*P. camemberti* CECT 2267	0.3	0.6
*P. expansum* CECT 2278	0.6	1.2
*P. roqueforti* CECT 2905	0.3	0.6
*P. digitatum* CECT 2954	1.2	2.3
*P. commune* CECT 20767	0.6	1.2
*P. solitum* CECT 20818	1.2	4.7
*P. verrucosum* VTT D-01847	0.6	1.2
*A. flavus* ITEM 8111	1.2	4.7
*A. parasiticus* CECT 2681	1.2	4.7
*A. ochraceus* CECT 2093	1.2	4.7
*A. lacticoffeatus* CECT 20581	1.2	2.3
*A. steynii* CECT 20510	2.3	9.4
*A. tubingensis* CECT 20543	1.2	4.7
*A. tubingensis* CECT 20544	1.2	18.8
*F. proliferatum* ITEM 12072	0.6	9.4
*F. verticillioides* ITEM 12052	0.6	2.3
*F. verticillioides* ITEM 12044	1.2	2.3
*F. graminearum* ITEM 126	4.7	9.4
*F. sporotrichioides* ITEM 12168	4.7	9.4
*F. poae* ITEM 9151	2.3	4.7

**Table 4:** The shelf life of bread loaves formulated with white mustard bran (MB) at 2.5, 5, 7.5, and 10 g/kg. The control did not receive treatment, and the commercial treatment was performed with sodium propionate (E-281) at 2 g/kg. The bread was contaminated with *P. commune* CECT 20767.

Treatment	Days						
	1	2	3	4	5	6	7
Control	−	−	−	+	++	+ +	++
Commercial	−	−	−	−	−	−	+
MB 2.5 g/kg	−	−	−	+	+ +	++	++
MB 5 g/kg	−	−	−	−	−	+	++
MB 7.5 g/kg	−	−	−	−	−	+	++
MB 10 g/kg	−	−	−	−	−	−	+

Results are expressed as follows: (−) means no superficial growth detected; (+) means superficial mycelium detected; (++) means superficial mycelium with spores detected.

## Data Availability

Data sharing not applicable.
